# Impact of Age and Duration of Symptoms on Surgical Outcome of Single-Level Microscopic Anterior Cervical Discectomy and Fusion in the Patients with Cervical Spondylotic Radiculopathy

**DOI:** 10.1155/2014/808596

**Published:** 2014-09-30

**Authors:** Farzad Omidi-Kashani, Ebrahim Ghayem Hasankhani, Reza Ghandehari

**Affiliations:** ^1^Orthopedic Research Center, Orthopedic Department, Imam Reza Hospital, Mashhad University of Medical Sciences, Mashhad 913791-3316, Iran; ^2^Faculty of Medicine, Mashhad University of Medical Sciences, Mashhad 913791-3316, Iran

## Abstract

We aim to evaluate the impact of age and duration of symptoms on surgical outcome of the patients with cervical spondylotic radiculopathy (CSR) who had been treated by single-level microscopic anterior cervical discectomy and fusion (ACDF). We retrospectively evaluated 68 patients (48 female and 20 male) with a mean age of 41.2 ± 4.3 (ranged from 24 to 72 years old) in our Orthopedic Department, Imam Reza Hospital. They were followed up for 31.25 ± 4.1 months (ranged from 25 to 65 months). Pain and disability were assessed by Visual Analogue Scale (VAS) and Neck Disability Index (NDI) questionnaires in preoperative and last follow-up visits. Functional outcome was eventually evaluated by Odom's criteria. Surgery could significantly improve pain and disability from preoperative 6.2 ± 1.4 and 22.2 ± 6.2 to 3.5 ± 2.0 and 8.7 ± 5.2 (1–21) at the last follow-up visit, respectively. Satisfactory outcomes were observed in 89.7%. Symptom duration of more and less than six months had no effect on surgical outcome, but the results showed a statistically significant difference in NDI improvement in favor of the patients aged more than 45 years (*P* = 0.032), although pain improvement was similar in the two groups.

## 1. Introduction 

Cervical spondylosis is a very common ailment [1]. These patients may present with regional cervical complaints, radiculopathy, or myelopathy [2]. These clinical manifestations existed alone or in combination with each other and most of the cases respond favorably to the conservative treatment [2, 3]. In refractory cases operative intervention may be considered. Although some authors believe that posterior foraminotomy with or without discectomy is applicable and preferred in some types of cervical disc herniation, the standard surgical technique is still anterior cervical discectomy and fusion (ACDF) [4–8].

While autogenous iliac crest bone graft is quoted to be the most common graft used autogenously throughout the world, nowadays most surgeons prefer not to use it to prevent its adverse short- and long-term morbidities [9]. Instead, commercial interbody cages with their various shapes and designs are commonly used. These spacers provide immediate structural stability and can be filled with various bone substitutes to promote interbody fusion [10, 11]. Anterior cervical plating can enhance the stability and fusion rate while at the same time reducing the possibility of cage subsidence, but in single-level cervical spondylotic radiculopathy (CSR) anterior plating is not usually necessary and not recommended [12–16].

Review of the literature indicates that there are lots of papers about epidemiology, natural history, various treatment options, and outcome of cervical disc herniation, but there are limited studies concerning the role of age and symptom duration in surgical results [17–19]. In this retrospective study we aim to evaluate the impact of these two important factors on outcome of the patients with single-level CSR who had been treated by microscopic ACDF.

## 2. Materials and Methods 

In this study, after local institutional review board approval (registration number 910106) we retrospectively evaluated our surgically treated patients with cervical spondylotic radiculopathy who had undergone single-level anterior cervical discectomy and fusion from August 2009 to January 2012 in our Orthopedic Department, Imam Reza Hospital, Mashhad, Iran. We included those cases with cervical radicular complaints due to cervical disc herniation refractory to at least six weeks of aggressive conservative treatment, neurologic deficit especially if clinically progressive, imaging and electrodiagnostic studies confirmed the clinical picture, operation was limited to simple one level anterior cervical discectomy and fusion with polyetheretherketone (PEEK) cage insertion and allogenic bone graft without any type of instrumentation, and those cases who had signed the informed consents. Our exclusion criteria comprised a follow-up period of less than two years, clinical and paraclinical findings more consistent with cervical spondylotic myelopathy (rather than radiculopathy), heavy smokers unable to stop smoking during the perioperative period, presence of more than one level of cervical disc herniation, underlying etiology other than disc herniation, those cases that the surgery was carried out without the assistance of microscopic equipment, and revision surgeries.

Preoperatively, the surgery and its probable advantages and disadvantages were explained to all the patients. Comprehensive historical and physical examinations were carried out and recorded. Duration of symptoms was calculated as the estimated period between the clinical manifestation and surgical operation. To assess the role of duration of symptoms, we placed our patients into two groups with six months as a border (group A with symptom duration less than 6 months and group B, vice versa). To evaluate the impact of age on surgical outcome, we set the age of 45 years as a threshold age for outcome.

The pain was measured subjectively and numerically on a sheet scaled from zero to ten (Visual Analogue Scale, VAS) [20]. The severity of disability was also assessed by Neck Disability Index (NDI) questionnaire [21]. Mousavi et al. in 2007 translated and validated the Iranian version of this questionnaire and it was proved to be completely reliable [22]. In this study we used its raw score (0 to 50) and did not express it as a percentage. Both questionnaires were completed again at the last followup of the patients.

### 2.1. Surgical Technique

After induction of general anesthesia, microscopic anterior cervical discectomy and fusion was accomplished according to standard technique previously reported by Kozak et al. and Husag [23, 24]. To achieve interbody fusion we used PEEK cage (Stryker PEEK Spacer Implant) filled with compacted allogenic chips bone graft (freeze dried cancellous allograft, Tissue Regeneration Corporation (TRC), Kish, Iran) without any additional plate fixation. After surgery, a Philadelphia collar was prescribed for about three to four weeks.

### 2.2. Postoperative Protocol

**Figure 1:**
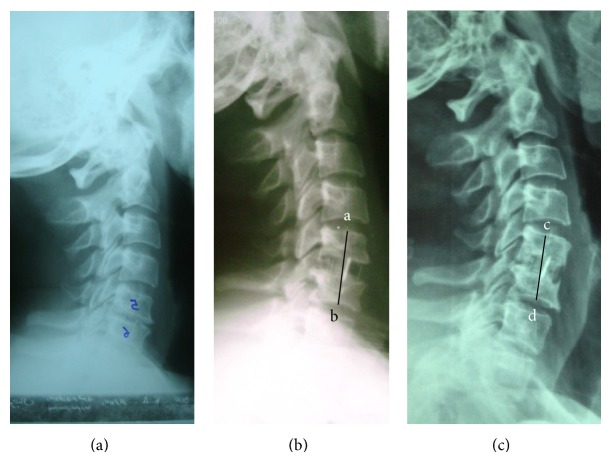
Radiographies are related to a 42-year-old female patient that presented with C5-C6 radiculopathy. ((a) Preoperative lateral view, (b) immediate postoperative view, and (c) view 41 months later.) When magnification is taken into account, 4 mm of cage subsidence (ab-cd) was found.

All significant intra- and postoperative complications were recorded appropriately. Radiologic assessment was repeated at 2 weeks, 3 months, and then annually, if required. Any radiological evidence denoting pseudoarthrosis, cage migration, or subsidence was also recorded. Pseudoarthrosis was suspected when the trabecular bone did not cross the adjacent vertebral bodies, more than 3° segmental motion on lateral dynamic views, or radiolucency more than fifty percent around the intended cage. We did not use computerized tomography routinely except in those cases with suspicious and symptomatic pseudoarthrosis. We defined cage subsidence as a reduction of more than 3 mm of the distance between midpoint of the upper endplate of the upper vertebral body and midpoint of the lower endplate of the lower vertebral body at the immediate and last follow-up visits (Figure 1).

Surgical outcome was assessed at the latest follow-up visit according to Odom's criteria and graded as excellent (when all preoperative symptoms were relieved and abnormal findings improved), good (when minimal persistence of preoperative symptoms existed and abnormal findings did not change or improved), fair (when definite relief of some preoperative symptoms happened but other symptoms did not change or slightly improved), and poor (when symptoms and signs did not change or were exacerbated) [25].

### 2.3. Statistical Analysis

Statistical analysis was performed by SPSS version 11.5 (SPSS Inc., Chicago, IL, USA). The Kolmogorov-Simonov test was used to assess normality of the data and paired *t*-test for comparison. We set statistical significance as a *P* value less than 0.05%.

## 3. Results 

We initially assumed that there were 74 patients who fulfilled our project's criteria but later six cases failed to be recruited; therefore, we could successfully evaluate and follow 68 patients: 48 (70.6%) female and 20 (29.4%) male. The mean age of the patients was 41.2 ± 4.3 (ranged from 24 to 72 years old). We could follow them up for 31.25 ± 4.1 months (ranged from 25 to 65 months). Involved levels included C5-C6 in 43 cases (63.2%), C6-C7 in 20 (29.4%), C4-C5 in 4 (5.9%), and C3-C4 in 1 (1.5%). Pre- and postoperative clinical indexes of our treated patients are shown in Table 1. This table shows that ACDF could significantly improve pain and disability in the patients with refractory radiculopathy.

Regarding six months of symptom duration, 48 cases were placed in group A while 20 cases were placed in group B (Table 2). Statistical analysis revealed that symptom duration of more than six months has no deleterious effect on surgical outcome of single-level ACDF in these radiculopathic patients. The results also showed a statistically significant difference in NDI improvement in favor of the patients aged more than 45 years (*P* = 0.032), although pain improvement was similar in the two groups.

**Figure 2:**
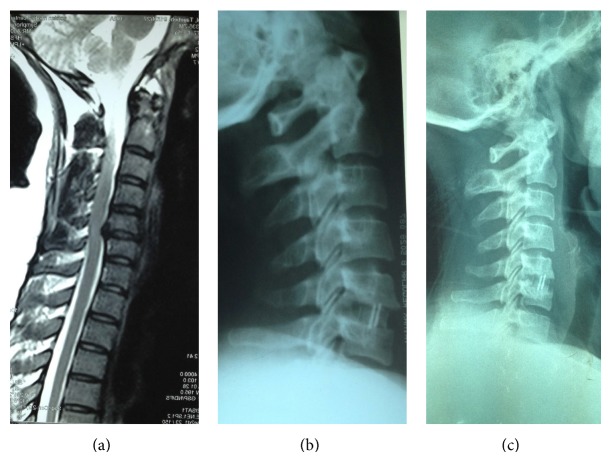
A 39-year-old female with C5-C6 cervical disc herniation (a). Lateral radiography on immediate postoperative day (b) was satisfactory but 26 months later frank pseudoarthrosis developed (c). The patient was completely asymptomatic.

Satisfactory outcomes were observed in 89.7% of the patients (Table 3), while successful fusion rate was 92.6%. There were cage subsidence in seven cases (10.3%), pseudoarthrosis in five (7.4%, Figure 2), superficial wound infections in five (7.4%), transient dysphonia in one (1.5%), and excessive intraoperative bleeding in one (1.5%). The latter happened due to inappropriate surgical approach (tissue dissection was carried out in lateral versus medial side of the carotid bundle and internal jugular vein was inadvertently injured). None of the patients with significant cage subsidence had fair or poor outcome but one of our patients with pseudoarthrosis was clinically symptomatic and finally treated with posterior cervical fusion and fixation. There was only one case that had both cage subsidence and pseudoarthrosis. This case was also clinically asymptomatic. Nonunion rate in the patients with cage subsidence and total cases was 14.3% and 7.4%, respectively. This indicates that cage subsidence can be a significant predisposing factor for pseudoarthrosis (*P* = 0.002).

## 4. Discussion

Although surgery is commonly used to treat the complaints of the patients with CSR, there are still many factors affecting the prognosis of these surgical procedures that should be evaluated. Some of known surgical prognostic factors that are frequently quoted include patient's psychiatric problem [26], failure to comply with a technically accurate surgery [2, 6], and smoking status of the patient [27] but menopausal status [28], workman's compensation claims [29], and cage subsidence [30] seem to have no adverse effect on clinical outcome of ACDF.

In previous decades anterior cervical discectomy was more prevalent but gradually, authors preferred to add intervertebral fusion to promote success. Gaetani et al. analyzed clinical outcomes of 153 cases with cervical spondylosis (including radiculopathy or myelopathy or both) that had been treated by anterior cervical discectomy alone [31]. They reported an excellent or good long-term outcome in 90.9% and 58.1% of the patients with radiculopathy and myelopathy, respectively. In their study, age, duration of symptoms (duration of complaints before diagnosis), and pathogenesis of disc herniation did not have significant effect on the surgical outcome, but presentation with pure radiculopathy was the most powerful positive predicting factor. In comparison, our study also confirmed that preoperative duration of the symptoms has no correlation with surgical outcome but unlike this study, we found that ACDF in the patients more than 45 years old can more potently improve pain and NDI (although this preference in pain improvement is not statistically significant). It should be noted that there is a difference between the two studies; all of our treated patients had CSR but a significant number of Gaetani's patients (28.7%) had myelopathy and this may be the key to this difference.

In the study conducted by Park et al., they evaluated the impact of menopause on bone fusion after single-level ACDF on 39 patients (11 in premenopausal and 28 in postmenopausal group) [28]. They could not find significant difference in the successful fusion rate or successful fusion type between the two groups. They reported a fusion rate of 90.9% in premenopausal and 89.2% in postmenopausal group. In the postmenopausal group, age and subsidence had significant adverse effect on successful fusion, and the prevalence of subsidence in the patients with a cage alone was higher than that in the cases with a plate fixation. In our study, we neither considered menopausal status nor used supplementary plating in our patients and overall fusion rate was 92.6% that was relatively comparable. We assessed the relation between age and clinical outcome (not fusion rate). Our results also confirmed that cage subsidence is a predisposing factor for developing pseudoarthrosis.

Yang and coauthors in a retrospective study on 38 patients (47 ACDFs) evaluated the subsidence and pseudoarthrosis using stand-alone PEEK cages filled with autogenous cancellous iliac bone graft [32]. They assigned major subsidence as a reduction in intervertebral height more than 3 millimeters and pseudoarthrosis as a change more than 2 millimeters in the interspinous distance on the flexion-extension lateral views. They observed major subsidence in 7 segments (14.9%) and found that small anteroposterior diameter of the cage and large intervertebral distraction were two important risk factors for cage subsidence. They also had seven pseudoarthroses (14.9%) that mainly belonged to two-level fusion group. Although we did not assess the relation between cage diameter, intervertebral distraction, and cage subsidence, we had seven (10.3%) and five (7.4%) patients with cage subsidence and pseudoarthrosis, respectively. The lower incidence of cage subsidence and nonunion in our study is probably due to the exclusion of two-level ACDF patients, because most of the pseudoarthrotic patients in Yang's study had two-level ACDFs.

Our study had several limitations. Aside from its retrospective design, several important factors were not taken into account. These included bone mineral density, menopausal status, workman's compensation claims, and, most importantly, routine postoperative computerized tomography scanning to verify bridging bone. We propose a prospective multicenter study with similar preoperative, operative, and postoperative protocols considering various risk factors affecting the outcomes.

## 5. Conclusion

According to this study, we conclude that single-level ACDF in surgical treatment of refractory patients with CSR is associated with long-term satisfactory outcomes. To achieve these acceptable results, duration of the symptoms seems to have no adverse effect but in the patients more than 45 years old, ACDF is more effective in improving disability and reducing pain, although the latter is not significant statistically.

## Figures and Tables

**Table 1:** Pre- and postoperative characteristics of our treated patients.

Indexes	Preoperative	Last followup	*P* value
NDI^†^	22.2 ± 6.2 (11–35)	8.7 ± 5.2 (1–21)	<0.001
VAS^‡^	6.2 ± 1.4 (4–10)	3.5 ± 2.0 (0–7)	<0.001

^†^NDI: Neck Disability Index.

^‡^VAS: Visual Analogue Scale.

**Table 2:** Role of duration of symptoms and age in surgical outcome of ACDF.

Index	Number	Preoperative		Improvement in last followup	
		NDI^†^	VAS^‡^	ΔNDI	ΔVAS
Duration of symptoms					
<6 months	48	21.8 ± 6.9	7.0 ± 1.8	13.0 ± 5.3	4.2 ± 2.0
>6 months	20	23.2 ± 4.2	6.2 ± 1.4	14.6 ± 9.6	2.7 ± 3.1
*P* value	—	0.536	0.110	0.865	0.101
Patients' age					
<45 years old	32	19.5 ± 5.2	6.0 ± 1.8	10.1 ± 9.5	3.1 ± 2.7
>45 years old	36	24.6 ± 6.1	7.4 ± 1.3	15.8 ± 4.6	4.3 ± 2.0
*P* value	—	0.204	0.09	0.032∗	0.130

^†^NDI: Neck Disability Index.

^‡^VAS: Visual Analogue Scale.

∗Significant statistically.

**Table 3:** Functional outcome according to Odom's criteria.

Functional outcome	Number	Percent
Excellent	40	58.8
Good	21	30.9
Fair	4	5.9
Poor	3	4.4
